# PTR*comb*iner: mining combinatorial regulation of gene expression from post-transcriptional interaction maps

**DOI:** 10.1186/1471-2164-15-304

**Published:** 2014-04-23

**Authors:** Gianluca Corrado, Toma Tebaldi, Giulio Bertamini, Fabrizio Costa, Alessandro Quattrone, Gabriella Viero, Andrea Passerini

**Affiliations:** 1 Department of Information Engineering and Computer Science (DISI), University of Trento, 38123 Trento, Italy; 2 Laboratory of Translational Genomics, Centre for Integrative Biology (CIBIO), University of Trento, 38123 Trento, Italy; 3 Bioinformatics Group, Department of Computer Science, Albert-Ludwigs-University Freiburg, 79110 Freiburg, Germany; 4 National Research Council, Institute of Biophysics, 38123 Trento, Italy

**Keywords:** Post-transcriptional regulation, Boolean matrix factorization, RNA binding protein (RBP), Binding site classification, Kernel machines, miRNA, Translation, CLIP

## Abstract

**Background:**

The progress in mapping RNA-protein and RNA-RNA interactions at the transcriptome-wide level paves the way to decipher possible combinatorial patterns embedded in post-transcriptional regulation of gene expression.

**Results:**

Here we propose an innovative computational tool to extract clusters of mRNA trans-acting co-regulators (RNA binding proteins and non-coding RNAs) from pairwise interaction annotations. In addition the tool allows to analyze the binding site similarity of co-regulators belonging to the same cluster, given their positional binding information. The tool has been tested on experimental collections of human and yeast interactions, identifying modules that coordinate functionally related messages.

**Conclusions:**

This tool is an original attempt to uncover combinatorial patterns using all the post-transcriptional interaction data available so far. PTR*comb*iner is available at http://disi.unitn.it/~passerini/software/PTRcombiner/.

## Background

Control of gene expression is a highly complex process involving the coordinated activity of multiple and heterogeneous biological factors. An underlying and intriguing general phenomenon is that biological molecules may act in a variety of different combinations to modulate cellular activities and to specifically react to changes in the biological milieu. To provide coordinated and multiple complex responses, several mechanisms are known to integrate a number of molecules in a combinatorial way.

Combinatorial post-translational modifications, such as methylation, acetylation, phosphorylation, and/or variations in regulatory trans-factors amounts, can influence the global regulation of gene expression at different levels. For example, the combinatorial epigenetic tagging of DNA and histones may enforce or reverse chromatin remodeling, thus playing a fundamental role in a variety of physiological and diseased cellular states [1,2]. Such combinatorial patterns of epigenomic modifications were found to be predictive of mRNA and ncRNA gene expression changes [3]. It is also well known that combinatorial control is important for transcription, where the interaction of transcription factors is critical for gene regulation [4]. The study of transcription factors combinatorics has been approached both on genome-wide [4-7] and element-specific scales [8,9]. As expected, the combinatorial arrangement of transcriptional regulation highly increases the overall possibilities to fine-tune the cellular response under different conditions. Out of the 23,000 genes encoded in the human genome, about 70% produce transcripts that are alternatively spliced. This yields multiple protein isoforms for each single pre-mRNA, increasing the variability of the proteome in eukaryotes. Cis-acting RNA sequence elements and enhancer complexes on pre-mRNA splicing are thought to form a combinatorial control network that allows exon recognition and splicing to occur [10,11]. Evidence of splicing combinatorial mechanisms can be obtained from the parallel analysis of several genes, but a complete picture of the combinatorial rules underlying the control of splicing is still lacking [12]. This is also true for the post-transcriptional control of gene expression, which is exerted by both cis-acting elements on the target mRNA and trans-factors, such as RNA binding proteins (RBPs) and non-coding RNAs (ncRNAs). The combined effect of trans-factors on mRNAs has been hypothesized to organize the so-called “post-transcriptional RNA operons (or regulons)” [13], but the global interplay of RBPs and ncRNAs on the same set of transcripts remains largely unexplored, despite being of paramount interest.

RBPs play an important role in all the processing stages of RNA fate, from synthesis to degradation. An essential step for functionally understanding RBPs is to identify their RNA substrates and the sites at which the interactions take place. The recent development of cross-linking and immunoprecipitation (CLIP) coupled to RNA-seq and related techniques has made it now possible to identify direct protein-RNA interactions in vivo at a very high base resolution [14,15]. These techniques provide positional information about the binding sites along the RNA sequence [16]. The combination of CLIP and RNA-seq provides an unparalleled capability to identify transcriptome-wide protein-RNA interactions. Modifications of the original method are also sprouting: the recent developments include iCLIP [15], PAR-CLIP, incorporating photoactivable bases in RNA [17], and CRAC, an affinity-tag protocol [18]. In 2012, a technique called global PAR-CLIP (gPAR-CLIP) was introduced, which allows the whole mRNA-bound proteome and its global occupancy profile to be identified [19]. The great progress in mapping protein-RNA interactions using genome-wide tools represents a fundamental source of information for post-transcriptional regulation of gene expression, but it does not specifically address possible combinatorial patterns embedded in RNA-protein and RNA-RNA interactions. Even if still largely incomplete, this binding site information creates for the first time a volume of data large enough to use for investigating possible combinatorial patterns of interactions by using machine learning techniques. Consequently, we can now start exploring post-transcriptional combinatorial rules in a systematic way.

To date, several tools have been developed to investigate and predict the interactions of transcription factors, mRNAs and miRNAs. Many of these bioinformatics approaches focus on predicting transcriptional networks by: i) modeling the expression level of a gene in terms of the predicted transcription factors that control its transcription rate [7,20,21]; ii) identifying clusters of co-regulated genes [22]; or, more generally iii) inferring portions of regulatory networks (see reviews by Li et al. [23] and Karlebach and Shamir [24]). Developing automated approaches to identify rules of combinatorial regulation at the post-transcriptional level would be of paramount interest to biologists. A few attempts have focused on analyzing miRNA-mediated interactions, by identifying putative feed-forward loops (FFLs), in which a transcription factor regulates a miRNA, and they together regulate a set of target genes [25-27], or miRNA-transcription factor motifs [28]. This relies on in silico target predictions coupled to gene expression data [29]. At a more general level, Krek et al. [30] developed PicTar, a combinatorial approach to predict the binding affinity of a pre-specified set of candidate miRNAs on a target mRNA by combining the output of individual miRNA target predictors. Coronello and Benos [31] refined the combinatorial model by integrating miRNA expression levels in the scoring function. Albeit limited to miRNA-mRNA interactions, these methods can be seen as initial attempts to uncover the combinatorial nature of post-transcriptional regulations at a genome-wide level. However, both methods require pre-specifying the set of miRNA to be checked, thus preventing their general applicability for mining novel unknown combinatorial patterns. The mining phase in fact requires an efficient search procedure to explore the space of possible combinatorial interactions, as a simple exhaustive enumeration of all combinations is computationally infeasible for all but the smallest sets of regulators.

Given the assumption that functionally related genes are more likely to be co-expressed, transcriptome data have been used to derive potential gene networks. Using a similar approach, inference of clusters of co-expressed genes and potential regulatory programs in post-translational controls have been developed. Building on the work by Segal et al. on learning module networks [32], Joshi et al. [33] developed a probabilistic approach to infer module networks in yeast, using both transcriptional and post-transcriptional regulators. The results obtained in this work generated interesting sets of hypotheses for regulatory pathways or processes in specific biological conditions (i.e. stress conditions). However, the method requires specific translational profiling time series as input.

Here we propose a method, called PTR*comb*iner, to study the combinatorial nature of post-transcriptional trans-factors. The method takes as input a collection of binding interactions and returns groups of factors sharing a conspicuous number of mRNA targets. It also analyzes the factors belonging to the same cluster in term of structural similarity of their binding sites. The identification of clusters is cast as a Boolean matrix factorization problem over the interaction matrix between trans-factors and mRNAs. This allows the simultaneous identification of multiple and possibly overlapping groups of factors that jointly cover as many interactions as possible. While Boolean matrix factorization has been employed in the data mining community to identify pattern sets, its application to the bioinformatics domain and especially to interaction data analysis is completely novel. Analyzing the trans-factors binding compatibility is cast as a binary classification problem aimed at discriminating between pairs of trans-factors in terms of their binding site similarity. The classifier employs state-of-the-art graph kernels [34] that account for predicted RNA secondary structure in addition to sequence information.

Thus, despite the still incomplete and noisy map of RNA-protein interactions, this Python-based tool will prove valuable in elucidating complex post-transcriptional networks.

## Results and discussion

**Figure 1:**
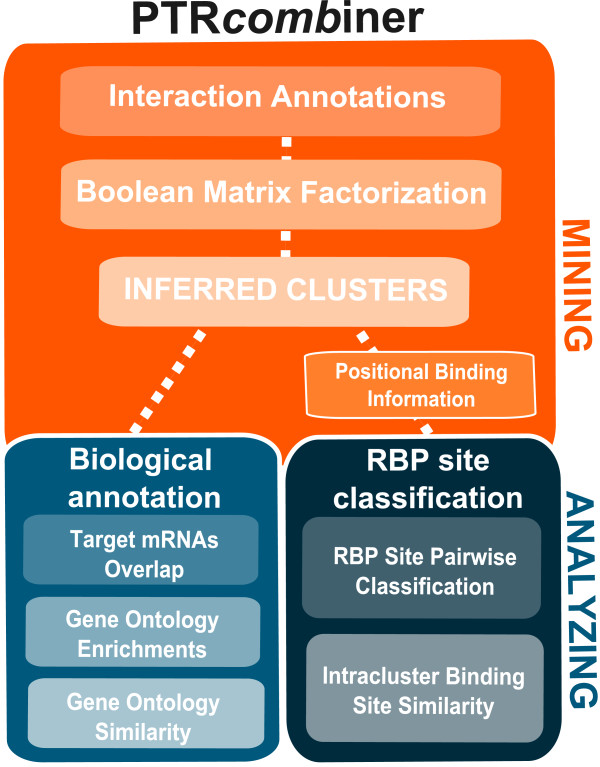
**PTR*comb*iner workflow scheme.** PTR*comb*iner is composed of two main methodological parts. The first, called “Mining combinatorial features” (orange panel), identifies groups (clusters) of regulatory elements (trans-factors) that act on common mRNAs. The second, called “Analyzing combinatorial features” (blue panels), explores the identified clusters to evaluate their biological characteristics in terms of commonly regulated mRNAs, Gene Ontology enrichments, and compatibility of binding sites among trans-factors belonging to the same cluster.

PTR*comb*iner (standing for *P*ost-*T*ranscriptional *R*egulation *comb*inatorial m*iner*) is a new tool composed of multiple modules that infer meaningful combinatorial relationships between mRNAs and their regulatory elements (trans-factors), namely RBPs and ncRNAs. This goal is achieved by extracting combinatorial information through a pattern-set mining approach and a meta-analysis of genome-wide data. PTR*comb*iner is divided into two activity components. The first, “mining combinatorial features” (orange panel in Figure 1), represents interaction data with a mathematical model. The model involves an approximate Boolean matrix factorization (BMF) of the interaction matrix, which identifies groups of regulatory elements acting on common mRNA UTRs, which we call clusters. The second, “analyzing combinatorial features” (blue panels in Figure 1), evaluates the biological characteristics of the clusters identified by the pattern-set miner. Each cluster is evaluated in terms of global biological meaning by Gene Ontology (GO) analysis over its mRNA targets and the binding site compatibility between the individual trans-factors in the cluster.

### Description of the dataset

The tool has been applied to the set of post-transcriptional interactions in human contained in the Atlas of UTR Regulatory Activity 2 (AURA 2) database (http://aura.science.unitn.it/, see [35], see Additional file 1). AURA 2 is a manually curated and comprehensive catalog of mRNA untranslated regions (UTRs) and their regulatory annotations, including interactions with trans-factors (mainly RBPs and miRNAs). To date, AURA 2 is the largest dataset of UTR-centered regulatory annotations taken from the whole range of existing experimental techniques, such as CLIP, RIP, SELEX, and RNAcompete. A subset of these techniques, namely CLIP and its variants followed by high-throughput sequencing, allows the positional annotation of the binding sites along the UTRs, while the others (RIP and its variants followed by microarray analysis or high-throughput sequencing) can only detect the presence of an interaction between a transcript and a trans-factor without the positional information of the specific binding site.

**Figure 2:**
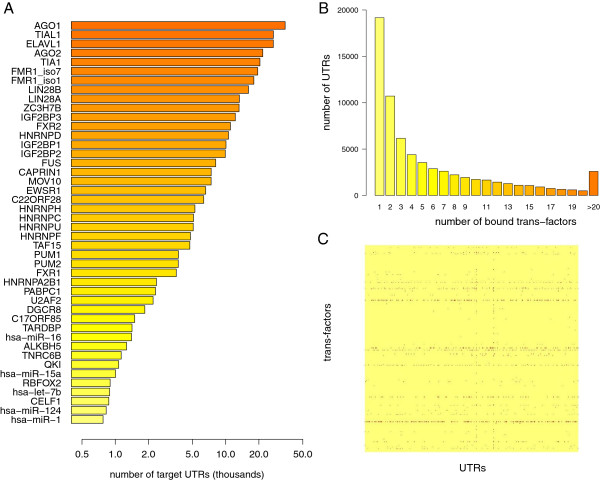
**Interaction maps annotated in AURA 2.(A)** Human trans-factors (RBPs and miRNAs) were ordered according to the number of annotated target UTRs. Trans-factors with less than 750 distinct UTR targets are not shown. **(B)** Distribution of the number of distinct trans-factors bound to the same UTR. **(C)** Graphical representation of the Boolean interaction matrix, derived from the input pairwise interactions. Each row corresponds to a trans-factor, each column to a UTR. Positive interactions are displayed in red.

As a working example for PTR*comb*iner, we considered the whole set of human interactions annotated in AURA 2. The available number of UTRs bound by each trans-factor ranges from 1 to 34,616, with median of 13 and a mean of 695 (trans-factors with more than 750 targets are displayed in Figure 2A). Symmetrically, the available number of trans-factors bound to the same UTR ranges from 1 to 64, with median 3 and mean 6 (the distribution is displayed in Figure 2B). Using these data, we built a Boolean matrix *C* with 67,962 rows (corresponding to the set of human UTRs, either 5’ or 3’, with at least one interaction) and 569 columns (corresponding to the set of annotated trans-factors, namely RBPs and miRNAs), where *C*_*ij*_ = 1 if the j^*th*^ trans-factor interacts with the i^*th*^ UTR, and *C*_*ij*_ = 0 otherwise (Figure 2C). The annotated interactions are collectively 395,395 (see Additional file 1), thus the sparsity of the interaction matrix is 0.01.

### Mining combinatorial features

After obtaining the interaction matrix, the first step of the analysis was to identify clusters of trans-factors (RBPs and/or miRNAs) that bind the same set of UTRs. Each of these clusters could be a candidate combinatorial member of a post-transcriptional regulatory code. This step thus aims to identify multiple overlapping clusters, which collectively cover most of the known interactions between trans-factors and UTRs. Boolean matrix factorization [36] provides this requirement by decomposing the matrix of known interactions (the Boolean matrix) in the product of two Boolean matrices. One of the matrices represents the clusters of the trans-factors, while the other, the UTRs in terms of their interactions within the clusters. The algorithm takes two arguments: the number of clusters to return (*k*), and a threshold (*τ*, ranging from 0 to 1). The *τ* value controls the minimal amount of shared UTRs inside a cluster. The higher the threshold, the more target UTRs should be shared among trans-factors in order for these to be considered as a cluster. The algorithm returns a list of clusters ordered by coverage, i.e. the number of interactions associated to each cluster (see Methods for a more detailed description).

**Figure 3:**
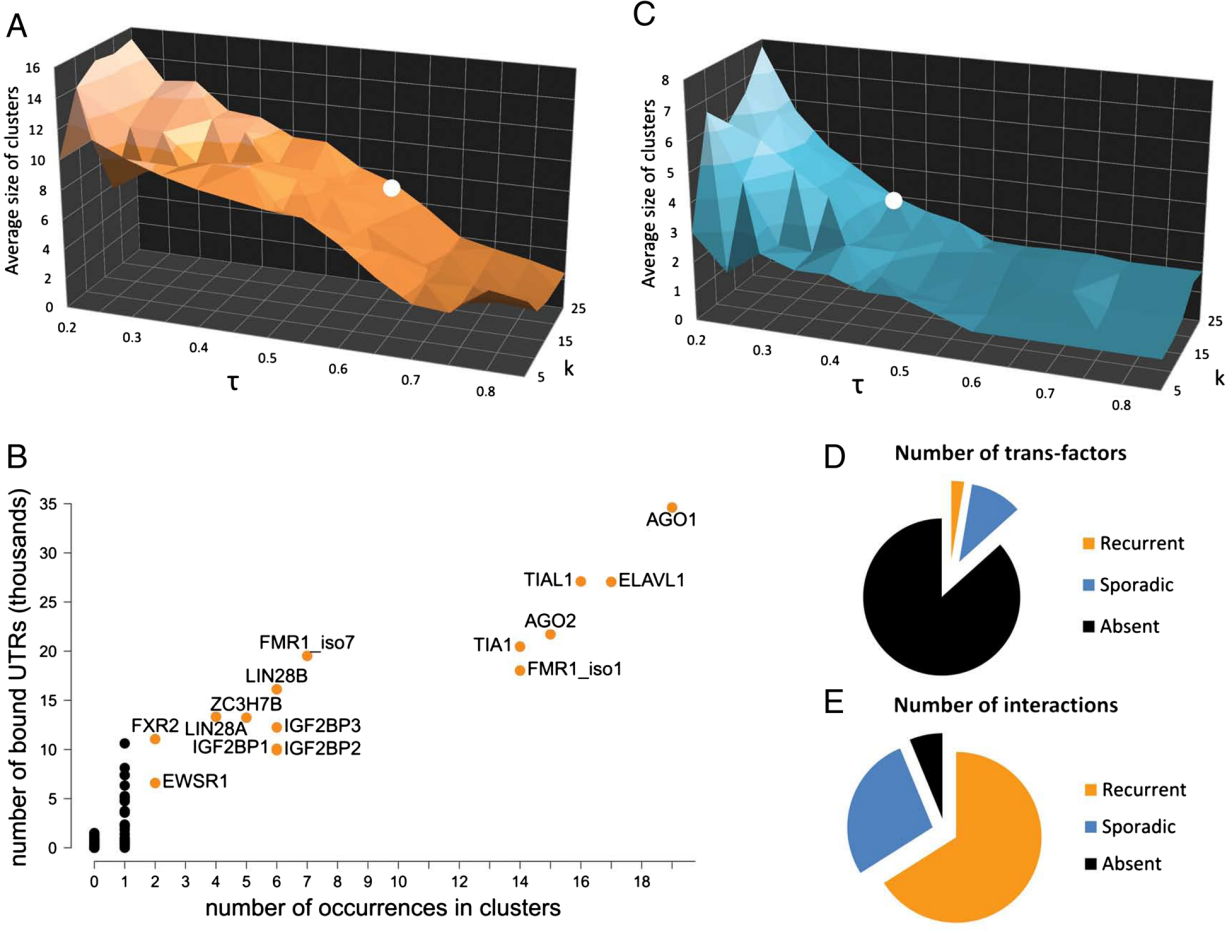
**Mining combinatorial features: identifying trans-factor clusters.** The average size (i.e. the number of trans-factors members) of the identified clusters is displayed at different combinations of *k* and *τ* values. **(A)** The white spot marks the configuration of the parameters selected to extract the clusters in the presence of recurrent trans-factors. **(B)** For each trans-factor, the number of occurrences in the identified clusters is plotted on the x axis, against the number of bound UTRs on the y axis. Recurrent trans-factors occurring in more than one cluster are labeled in orange. **(C)** After all the recurrent trans-factors were removed from the analysis, the average size of the identified clusters was displayed on the y axis at different combinations of *k* and *τ* values. The white spot identifies the configuration of parameters selected to extract the clusters of sporadic trans-factors. **(D)** The proportions among the number of recurrent, sporadic, and absent trans-factors are shown. **(E)** The proportions among the number of interactions associated with recurrent, sporadic, and absent trans-factors are shown.

To analyze the behavior of the algorithm when varying its parameters, we produced a surface parameter graph (Figure 3A) where the average number of trans-factors belonging to each cluster (cluster size) was calculated using various combinations of *k* and *τ* values (see Additional file 2). Given a certain *τ*, different values of *k* do not affect the average cluster size, which appears to be mainly *τ* dependent. We chose the *τ* value giving an average cluster size equal to the average number of trans-factors bound to a single UTR (Figure 2C). We thus considered a *τ* of 0.6, resulting in clusters composed of an average of six trans-factors. Interestingly, this value points to a stable region of the *k* and *τ* surface, in which the number of trans-factors for each cluster does not change drastically in the surrounding area (Figure 3A, circle). Table 1 shows the clusters obtained with the selected threshold. The first nine clusters are composed exclusively of RBPs, as well as the clusters R11 to R19, R22 and R25. The first cluster displaying co-occurrence of RBPs and miRNAs was R10, followed by clusters R20, R21, R23 and R24. No cluster uniquely composed of miRNAs was present. We would like to stress that 5 out of 25 clusters do not represent real combinations, as they comprise only one trans-factor. We refer to these one-element clusters as “singletons”. Recalling that the algorithm aims to cover as many interactions as possible with the set of clusters, a singleton can be extracted by the algorithm whenever a trans-factor has many interactions that are not shared with any other trans-factor for which experimental interaction data are available.

**Table 1:** List of the inferred clusters in the presence of recurrent trans-factors

**Class**	**Cluster**	**Trans-factors**
RBP	Clust R01	AGO1, AGO2, ELAVL1, FMR1_iso1, FMR1_iso7,
		FXR2, LIN28A, LIN28B, MOV10, TIA1, TIAL1,
		ZC3H7B
RBP	Clust R02	AGO1, AGO2, ELAVL1, IGF2BP1, IGF2BP2,
		IGF2BP3, TIAL1
Singleton	Clust R03	AGO1
RBP	Clust R04	ELAVL1, HNRNPD
RBP	Clust R05	AGO1, AGO2, ELAVL1, EWSR1, FMR1_iso1,
		FUS, LIN28A, LIN28B, TAF15, TIA1, TIAL1,
		ZC3H7B
RBP	Clust R06	AGO1, ELAVL1, TIA1, TIAL1
RBP	Clust R07	AGO1, FMR1_iso1, FMR1_iso7
RBP	Clust R08	AGO1, AGO2, CAPRIN1, ELAVL1, FMR1_iso1,
		FMR1_iso7, LIN28B, TIA1, TIAL1, ZC3H7B
RBP	Clust R09	AGO1, AGO2, C22ORF28, ELAVL1, FMR1_iso1,
		FMR1_iso7, LIN28B, TIA1, TIAL1, ZC3H7B
RBP-miRNA	Clust R10	LIN28A, LIN28B, hsa-miR-221*
RBP	Clust R11	AGO1, HNRNPH
RBP	Clust R12	AGO1, AGO2, ELAVL1, FMR1_iso1, HNRNPC,
		TIA1, TIAL1
Singleton	Clust R13	PUM1
RBP	Clust R14	AGO1, AGO2, ELAVL1, FMR1_iso1, FMR1_iso7,
		HNRNPU, TIA1, TIAL1
RBP	Clust R15	AGO1, AGO2, ELAVL1, FMR1_iso1, FMR1_iso7,
		HNRNPF, TIA1, TIAL1
RBP	Clust R16	AGO1, AGO2, ELAVL1, EWSR1, FMR1_iso1,
		FMR1_iso7, FXR1, FXR2, LIN28A, LIN28B, TIA1,
		TIAL1, ZC3H7B
RBP	Clust R17	AGO1, AGO2, ELAVL1, FMR1_iso1, IGF2BP1,
		IGF2BP2, IGF2BP3, PUM2, TIA1, TIAL1
Singleton	Clust R18	PABPC1
Singleton	Clust R19	U2AF2
RBP-miRNA	Clust R20	AGO1, AGO2, ELAVL1, FMR1_iso1, IGF2BP1,
		IGF2BP2, IGF2BP3, TIA1, TIAL1, hsa-miR-130a,
		hsa-miR-130b, hsa-miR-148a, hsa-miR-148b,
		hsa-miR-301a, hsa-miR-301b
RBP-miRNA	Clust R21	AGO1, AGO2, ELAVL1, FMR1_iso1, IGF2BP1,
		IGF2BP2, IGF2BP3, TIA1, TIAL1, hsa-miR-15a,
		hsa-miR-15b, hsa-miR-16, hsa-miR-424
Singleton	Clust R22	DGCR8
RBP-miRNA	Clust R23	AGO1, AGO2, ELAVL1, FMR1_iso1, IGF2BP1,
		IGF2BP2, IGF2BP3, TIA1, TIAL1, hsa-miR-106b,
		hsa-miR-17, hsa-miR-20a, hsa-miR-320,
		hsa-miR-93
RBP-miRNA	Clust R24	AGO1, AGO2, ELAVL1, IGF2BP1, IGF2BP2,
		IGF2BP3, TIAL1, hsa-let-7a, hsa-let-7b,
		hsa-let-7c, hsa-let-7d, hsa-let-7e, hsa-let-7f,
		hsa-let-7g, hsa-let-7i
RBP	Clust R25	AGO1, AGO2, ELAVL1, FMR1_iso1,
		HNRNPA2B1, TIA1, TIAL1

Clusters were classified according to their composition: “RBP” for those composed exclusively of RBPs; “miRNA”, composed exclusively of miRNAs; “RBP-miRNA”, composed of both RBPs and miRNAs; and “Singleton”, composed of only one trans-factor.

As detailed in the previous section, different trans-factors have highly different numbers of annotated interacting UTRs. Being driven by coverage, the algorithm is inherently biased towards clusters of “widely interacting” trans-factors. Therefore, when we analyzed the composition of the clusters, we observed that some trans-factors are present in multiple clusters. For example, the Argonaute proteins AGO1 and AGO2, and the well-known RBP ELAVL1/HuR, occur in 19, 15 and 17 out of 25 clusters, respectively. AGO1 and AGO2 are components of the RNA-induced silencing complex (RISC), the protein complex which is responsible for down-regulating mRNAs [37]. These proteins bind different classes of small ncRNAs, such as miRNAs and small interfering RNAs (siRNAs), leading the Argonaute proteins to their specific targets through sequence complementarity, thus silencing their targets. Therefore, it is not surprising to find AGO1 and AGO2 in almost all of the clusters, given the widespread activity of these proteins. Moreover, in accordance with our results, AGO1 and AGO2 have been found to interact with ELAVL1/HuR [38]. Despite this interaction, the two proteins avoid any binding overlap on the target mRNAs: AGO proteins preferentially bind the boundaries of UTRs, while ELAVL1/HuR binds uniformly along UTRs and disappears toward the stop codon and the polyadenylation site [39]. ELAVL1/HuR, a member of the ELAV family, is known to be broadly expressed in tissues and to bind AU-rich elements in the 3’ UTRs of thousands of mRNAs [39-41]. Moreover, it has been demonstrated that ELAVL1/HuR displays competitive and cooperative interactions with miRNAs/RISC [42,43], that these interactions may depend on the proximity between the protein and miRNA binding sites [39,44] and that it is part of a complex mRNA network for coordinating gene expression [45]. These results are in agreement with the cluster composition in Table 1 and support our finding that the elements displaying the highest number of interactions (orange dots in Figure 3B) are those that most frequently occur across the clusters. For this reason, we called these elements “recurrent trans-factors” and these clusters “Rk” (R standing for recurrent and k standing for cluster number and ranging from 1 to 25).

To explore trans-factors that have a narrower spectra of interactions and thus occur less frequently in the clusters, we removed all recurrent trans-factors (i.e. those found in more than one cluster) and ran a second iteration of the algorithm. This second iteration focused on trans-factors that appeared in none or only one of the clusters of the previous analysis. We termed these trans-factors “sporadic”. Again, we studied the behavior of the algorithm when the *k* and *τ* parameters were varied, in order to select the most appropriate combination of values (see Additional file 3). In this case, the best choice of *τ* was 0.4 (Figure 3C). This value returned clusters comprised of an average number of three trans-factors, which corresponds to the average number of sporadic elements bound to each UTR. We called these clusters “Sk” (S standing for sporadic and k standing for cluster number and ranging from 1 to 25) (Table 2). It is clear that the majority of the clusters (15 out of 25) are singletons. In contrast to results obtained when recurrent factors were included, we observed that four clusters were formed exclusively by miRNAs (namely, S09, S14, S16 and S22). Interestingly, PUM2, which was also found as a member of the recurrent clusters, formed two distinct clusters with different sets of miRNAs (S10 and S21). In line with this, some evidence has suggested that PUM2 associates with miRNAs. PUM2 is known to act as a translational repressor in several organisms, contributing to dendritic RNA localization and silencing [46] and regulating synaptic formation [47]. Moreover, an extensive interaction between PUM1 and PUM2 with the miRNA regulatory system has been suggested [48], indicating that interactions between the RBPs and the miRNAs in translational regulation may be more frequent than previously thought. In addition, a recent computational analysis suggested that specific groups of miRNA binding sites localize within 50 nt from PUM2 binding sites, giving support to a possible cooperativity between PUM2 and miRNA in mRNA degradation [49] and to the biological meaning of our clusters where PUM2 acts on mRNAs in combination with different miRNAs. In particular, hsa-miR-221 and hsa-miR-222, which are part of cluster S21 together with PUM2, appear to colocalize with this RBP [49].

**Table 2:** List of the inferred clusters composed of sporadic trans-factors

**Class**	**Cluster**	**Trans-factors**
Singleton	Clust S01	HNRNPD
RBP	Clust S02	CAPRIN1, FUS, FXR1, MOV10, TAF15
Singleton	Clust S03	HNRNPH
RBP	Clust S04	C22ORF28, CAPRIN1, MOV10
Singleton	Clust S05	HNRNPC
Singleton	Clust S06	HNRNPU
Singleton	Clust S07	HNRNPF
Singleton	Clust S08	PUM1
miRNA	Clust S09	hsa-miR-15a, hsa-miR-15b, hsa-miR-16,
		hsa-miR-424
RBP-miRNA	Clust S10	PUM2, hsa-miR-130a, hsa-miR-130b,
		hsa-miR-148a, hsa-miR-148b, hsa-miR-19a,
		hsa-miR-19b, hsa-miR-301a, hsa-miR-301b
Singleton	Clust S11	HNRNPA2B1
Singleton	Clust S12	PABPC1
Singleton	Clust S13	U2AF2
miRNA	Clust S14	hsa-miR-106b, hsa-miR-17, hsa-miR-20a,
		hsa-miR-93
RBP	Clust S15	MOV10, PUM2
miRNA	Clust S16	hsa-let-7a, hsa-let-7b, hsa-let-7c, hsa-let-7d,
		hsa-let-7e, hsa-let-7f, hsa-let-7g, hsa-let-7i
Singleton	Clust S17	DGCR8
Singleton	Clust S18	C17ORF85
Singleton	Clust S19	TARDBP
RBP	Clust S20	FUS, MOV10, TAF15
RBP-miRNA	Clust S21	PUM2, hsa-miR-103, hsa-miR-107,
		hsa-miR-183, hsa-miR-221, hsa-miR-222,
		hsa-miR-23b, hsa-miR-25, hsa-miR-27a,
		hsa-miR-27b, hsa-miR-32, hsa-miR-92a,
		hsa-miR-96
miRNA	Clust S22	hsa-miR-103, hsa-miR-107, hsa-miR-15a,
		hsa-miR-15b, hsa-miR-16, hsa-miR-29a,
		hsa-miR-29b, hsa-miR-29c, hsa-miR-424
Singleton	Clust S23	CELF1
Singleton	Clust S24	hsa-miR-124
Singleton	Clust S25	hsa-miR-1

Clusters were classified according to their composition, as for Table 1.

Despite that only a small fraction of known trans-factors occur in the clusters (Figure 3D), the vast majority of the existing interactions is covered by the identified clusters (Figure 3E). The fact that the most of the trans-factors are not part of any cluster is due to the paucity of available information, i.e. the overall number of experimental data obtained until now for many of the trans-factors is still far from being complete. This is not surprising given the novelty of the experimental techniques involved. It is clear that more data are needed to obtain a reliable and exhaustive description of all the possible combinatorial interactions in human. The availability in the next future of high-throughput experimental data covering an increasingly larger amount of trans-factors will open up new possibilities to uncover new and more reliable clusters and to obtain new combinatorial information.

### Analyzing combinatorial features - step 1: biological annotation

The first step in analyzing the clusters obtained from the mining module was to measure how much they differ in terms of: a) target mRNAs (Target mRNAs Overlap module, Figure 1); b) enriched ontological terms (biological process, molecular function, and cellular component in Gene Ontology Enrichments module, Figure 1); and c) similarity among ontological terms (Gene Ontology Similarity module, Figure 1).

**Figure 4:**
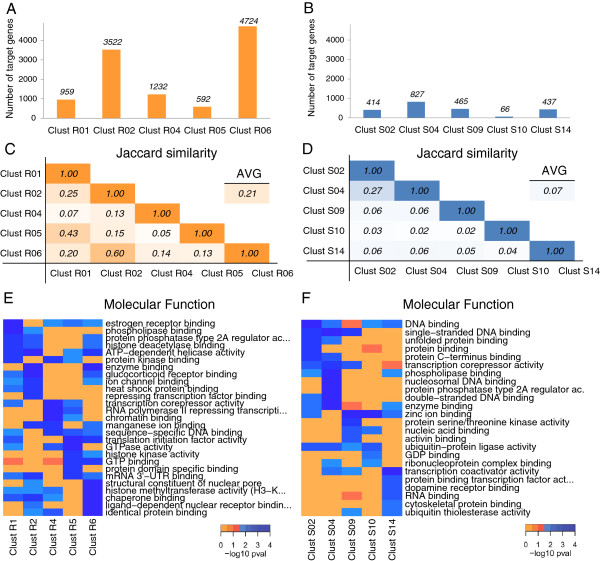
**Analyzing combinatorial features, step 1: biological annotation of the trans-factor clusters.** The number of HGNC genes targeted by the top five ranking clusters that included either recurrent trans-factors **(A)** or sporadic trans-factors **(B)** are displayed. The Jaccard similarities among the top five ranking clusters that included recurrent trans-factors **(C)** or sporadic trans-factors **(D)** are given. Heatmap showing the top enriched Molecular Function GO terms associated to the lists of genes targeted by the top five ranking clusters obtained with recurrent trans-factors **(E)** or with sporadic trans-factors **(F)** are shown. Singletons were excluded from the analyses.

Overall, we expected to obtain a large amount of regulated genes that form clusters in the presence of recurrent trans-factors from the mining module, because they display the highest number of annotated interactions (Figure 3B). In fact, the module Target mRNAs Overlap revealed that several hundred genes were co-regulated by recurrent trans-factors (Figure 4A and Additional file 4). The average number of HGNC genes regulated by the first five clusters (excluding singletons) was 2,206, ranging from 592 for cluster R05 to 4,724 for cluster R06. The number of target genes was markedly reduced in the clusters of sporadic trans-factors, as expected given their greater specificity (Figure 4B and Additional file 5). The average number of HGNC genes regulated by the first five clusters was 442, ranging from 66 for cluster S10 to 827 for cluster S04.

We then explored the possibility of finding the same mRNA targets in different clusters, by calculating the overlap among the populations of genes grouped in different clusters using the Jaccard similarity (see Methods). For the clusters including recurrent trans-factors, the overlap was 21% on average (Figure 4C). This result suggests that the method is able to group sets of specific target mRNAs even if they share common trans-factors. However, some leakage is present: for example, cluster R01 and cluster R05 share 43% of their targets. This is not surprising as careful inspection of the elements forming these clusters revealed that R01 and R05 share seven RBPs and are individually characterized only by FXR1 and FUS, respectively. The same is observed for clusters R02 and R06, which share 60% of their targets. In this case, cluster R06 shares with R02 almost all its own trans-factors (AGO1, ELAVL1, and TIAL1). Focusing on clusters of sporadic elements revealed that the average overlap decreased to 7%, one-third of that from the previous analysis (Figure 4D). In this case, the maximum overlap was between clusters S02 and S04, which share 27% of their targets. Both of these clusters comprise CAPRIN1 and MOV10, while they do not share FUS, FXR1, TAF15 and C22ORF28 (see Table 2). These results confirm the effectiveness of the repeated run of analysis in identifying distinct, small-sized sets of genes, uniquely regulated by specific sets of sporadic trans-factors.

The Gene Ontology Enrichment module of the tool allowed us to study the biological relevance of the mined clusters. The module attempts to identify parts of common and biologically coordinated mechanisms or processes that govern coherent cellular outcomes and that could be characterized by the identified groups of preferential interactions. In this module, the analysis is expanded from single genes to more general biological annotations, allowing the inferred clusters of trans-factors to be compared by the gene ontology (GO) enrichment analysis of their target mRNAs. An initial and effective way to compare enrichments is to display the top enriched GO terms for each cluster. Such a comparison is shown in Figure 4E for the top five non-singleton Rk clusters, using the molecular function (MF) branch of GO as example (see Additional file 6 for all the enrichment results). The modular blocks of enriched terms scattered along the columns of the heatmap clearly indicate that the clusters have marked and distinct molecular function consensuses. Similarly to what we observed for the mining module results, the modularity of the enrichments was further reinforced in the top five non singleton Sk clusters (Figure 4F and Additional file 7). Here, the occurrence of terms enriched in multiple clusters is an infrequent event. Clusters S02 and S04 share the most similar enrichment signature, mirroring the strong similarity observed between the two clusters (see above and Figure 4D).

**Figure 5:**
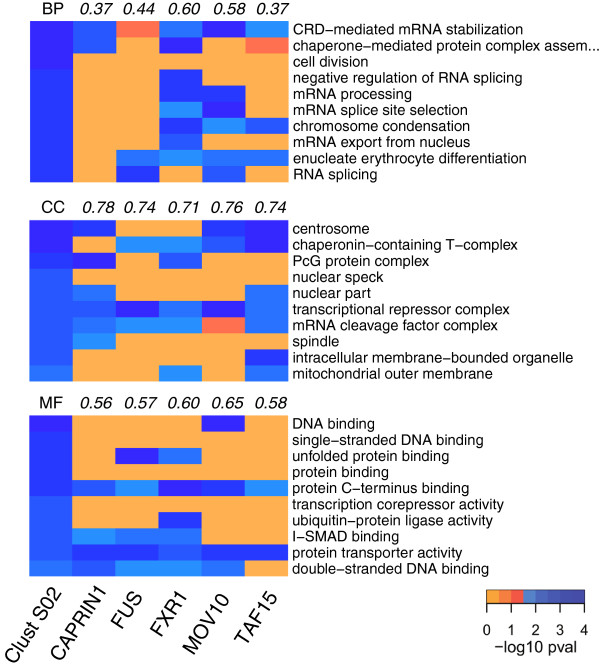
**Analyzing combinatorial features, step 1: intra-cluster enrichment analysis.** For cluster S2, the first column of the heatmap displays the top enriched GO terms associated to its target mRNAs, with the GO terms biological products (BP) in the upper panel, cellular components (CC) in the middle panel, and molecular functions (MF) in the lower panel. The remaining columns show the enrichment analysis performed on the lists of mRNAs interacting with each individual member of cluster S2. Cells are colored according to the enrichment P values, with significant enrichments displayed in shades of blue. The top rows of each panel list the semantic similarity values between enriched terms associated to the cluster and those associated to the single trans-factors.

After comparing the enrichment of different clusters, we next assessed how the ontological enrichment of genes regulated by one cluster differs from the ontological enrichments of genes regulated by the individual trans-factors forming the cluster. This intra-cluster comparison allowed us to potentially identify “emergent enrichments”, i.e. to detect GO terms enriched exclusively in a set of genes regulated by a set of trans-factors forming a cluster. An example of this analysis is shown for cluster S02 (Figure 5). The target genes in this clusters were specifically enriched for the biological process (BP) term “cell division”, the cellular component (CC) term “nuclear speck”, and the molecular function (MF) term “transcription corepressor activity”. These results strongly suggest that the clusters have emergent and specific combinatorial properties, as expected for combinatorial mechanisms.

To further estimate the similarity between the ontological enrichments associated with each cluster, the tool is able to calculate the semantic similarity values, which account for semantic similarity relationships between non-identical ontological terms (see Methods). The module Gene Ontology Similarity calculates the semantic similarity values for all the three branches of GO and between both each pair of clusters (inter-cluster semantic similarity) and the single trans-factors belonging to the same cluster (intra-cluster semantic similarity). In Figure 5, the top rows of each panel list the semantic similarity values between enriched terms associated to single trans-factors and enriched terms associated to cluster S02. Here, the stronger semantic similarities are observed among the CC enrichments, while the weakest values lie among the BP enrichments. This analysis can also help to rank the ontological distance between a cluster and its trans-factors according to the semantic similarity values. For example, FXR1 globally shows the highest similarity with the enrichment of cluster S02 (Figure 5).

### Analyzing combinatorial features - step 2: classifying RBP-binding sites

To suggest some potential binding mechanisms based on the available experimental data [35], the second step of the analysis focuses on the RBPs forming the previously identified clusters. The underlying idea is that whenever two RBPs (in the same cluster) are characterized by similar binding sites over the mRNA, then a concurrent binding, either competitive or cooperative, has occurred.

Although experimental methods (e.g., CLIP-seq) can be used to gather information about the proximity of binding sites for specific RBPs, the resulting information is corrupted by several types of noise sources: 1) a considerable fraction of binding sites can remain undetected (false negatives) because the methods are applied on cells of a particular type and in specific growth conditions, in which not all the potential bound mRNAs are expressed; 2) interactions that are transient can be mistakenly identified as stable (false positives); and 3) post-processing analysis, such as mapping and peak detection, can increase the number of false negatives due to the difficulty of dealing with splice sites and the stringent thresholds needed for a confident detection, respectively. Computational models for RBP target detection are therefore valuable tools in dealing with the low signal-to-noise ratio of current experimental techniques. Such models can significantly increase the precision with which target sites are resolved and can uncover sites that would be otherwise missed by experimental protocols. To determine if two RBPs are are likely to interact in a cooperative or competitive fashion, we first compiled an equivalent in silico model of the preferred RBP target sites. Given a cluster of RBPs and their binding sites, the module trains a machine learning algorithm to discriminate between binding sites of two different RBPs, for all possible pairwise combinations of proteins belonging to the same cluster. Two proteins are likely to have different binding sites when the algorithm effectively distinguishes between their binding areas. In contrast, compatible binding sites are more likely to lead to a difficult discrimination task. Discrimination is based on a kernel machine binary classifier [50] capable of computing similarity between base sequences in terms of their respective putative secondary structures [34] and thus of the spatial conformation of their binding sites (see Methods for a detailed description of the classifier). The structural component of this discrimination has a strong biological significance, since RNA interactions are not exclusively driven by sequence specificities.

**Figure 6:**
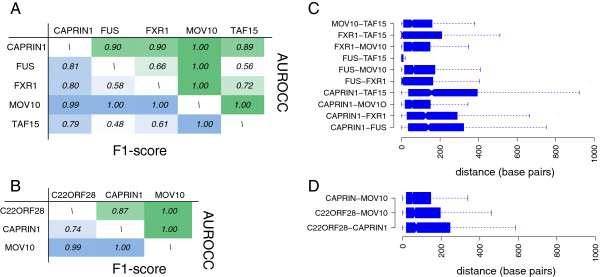
**Analyzing combinatorial features, step 2: RBP site classification.** The pairwise classification performance values are displayed for cluster S2 **(A)** and cluster S4 **(B)**. AUROCC values are shown in the top-right halves of the tables and colored in shades of green, while F1-scores are shown in the bottom-left halves of the tables and colored in shades of blue. The distributions of the distances between binding sites of two distinct trans-factors belonging to cluster S2 **(C)** and S4 **(D)** are also shown.

We report the results of the classification analysis performed on the first two clusters of sporadic trans-factors (excluding singletons), namely, the cluster S02 that comprises CAPRIN1, FUS, FXR1, MOV10, and TAF15, and the cluster S04 that comprises C22ORF28, CAPRIN1, and MOV10 (see Table 2). For each RBP, we randomly selected 2,500 mRNA UTR stretches (of 20–70 nt) from available binding coordinates of large-scale experiments (CLIP-seq and related techniques) stored in the AURA 2 database (see Additional file 8). The classification performances for clusters S02 and S04 are displayed in Figure 6A and Figure 6B, respectively. Performance is computed with the AUROCC and F1-score measures. AUROCC is an aggregate measure that evaluates the quality of a classifier when varying the threshold, to decide when a prediction should be considered positive. An AUROCC value of 0.5 corresponds to a random predictor, while an AUROCC value of 1 indicates perfect discrimination. The F1 score is the harmonic mean between precision and sensitivity, trading off the two complementary measures (see Methods for the detailed explanation of these performance measures). Considering the cluster S02, the classifier discriminates the binding sites of only a subset of the RBPs in the cluster (Figure 6A). The most specific binding sites were observed for CAPRIN1 (with an average AUROCC of 0.92 and an average F1 of 0.85) and MOV10 (with an average AUROCC and F1 of 1.0). FUS and TAF15 seem to have more compatible binding sites. An AUROCC of 0.56 is in fact very close to the performance of a random predictor, suggesting that these proteins share a similar if not identical set of binding sites. Indeed, these two proteins are known paralogues, both belonging to the FET family of RNA-binding proteins [51]. Higher AUROCC values were achieved when distinguishing the binding sites of FXR1 from those of FUS and TAF15 (0.66 and 0.72, respectively), and maximal AUROCC values were found for MOV10, suggesting different binding sites. Classification performances of cluster S04, displayed in Figure 6B, were generally high, suggesting that the UTR stretches that are bound by the RBPs forming the cluster (C22ORF28, CAPRIN1 and MOV10) are different. Figures 6C and 6D show the distribution of the distances between couples of binding sites lying on the same mRNA and targeted by distinct RBPs, for cluster S02 and S04 respectively. The distances between the binding sites of FUS and TAF15 are much lower than those of the other distributions, indicating that the two proteins tend to bind to the same or very close regions. The average distance between FXR1 and FUS or TAF15 is also low, but it has a much larger spread. A large average distance is observed for the other cases and indicates a high discrimination capacity of the classifier.

These examples show how we can start to use in silico modeling of RBPs interactions to investigate their collaborative and/or competitive effects. This type of modeling is effective when the experimental data is affected by noise, since it recovers missed interactions (false negatives) and filters out accidental interactions (false positives). A related predictive system was used in [52], where it was shown that a model trained on a set of AGO2 HITS-CLIP sites could effectively identify binding sites missed by the high-throughput experiment. These findings have been verified comparing the predicted AGO2 targets with changes in transcript expression levels upon AGO2 knockdown. Conversely this type of trend was not observed for the original HITS-CLIP-detected sites indicating a significant impact of false negatives in the experimental setting. The upgrade to predictive models capable of this level of accuracy will allow more sophisticated investigations, such as those warranted by the combinatorial analysis presented in this work. For instance, a competitive effect can be hypothesized if two RBPs, found in the same cluster, exhibit a compatible preference for the same target region, even when the experimental data are incomplete and do not report overlapping interaction areas. Conversely, if the target regions are predicted to be sufficiently close but not overlapping, a cooperative effect can be hypothesized, even when experimental data cannot resolve the distinct areas and these are therefore reported as overlapping.

## Alternative workflows

### 1 - Filtering interactions by experimental technique

PTR*comb*iner allows a selection of the source interaction data to be use, thereby taking into account the different experimental approaches currently available for mapping protein-RNA interactions [16] and producing technically homogeneous results. This method-selection feature of PTR*comb*iner can be employed to exclude from the analysis the interactions obtained from techniques that are considered more unreliable or inopportune for specific analyses. As an example of the method-selection tool, we filtered the human interactions annotated in AURA 2 according to the evidence type, identifying three subsets of the original dataset. The first subset considered only *PAR-CLIP* experiments and includes a total of 28 trans-factors (all RBPs) and 44,445 UTRs, with an average number of interactions per UTR of 4.64 (206,065 annotated interactions). The second subset considered all the *other CLIP* experiments, namely CLIP, CLIP-seq, HITS-CLIP and iCLIP. It includes a total of 12 trans-factors (all RBPs) and 45,478 UTRs, with an average number of interactions per UTR of 2.39 (108,528 annotated interactions). The third subset used only interactions found by RNA immunoprecipitation *RIP*. To date, it includes a total of 22 trans-factors (all RBPs) and 21,951 UTRs, with an average number of interactions per UTR of 1.4 (30,755 annotated interactions).

In the first subset (PAR-CLIP), we observed the presence of clusters composed exclusively of RBP (summarized in the Additional file 9: Table S1). The clusters are similar to those observed when the whole set of interactions was analyzed, excluding trans-factors with none or few interactions based on PAR-CLIP (e.g., for AGO1, only one-tenth of annotated interactions originated from PAR-CLIP experiments). Considering only the RBP interactions obtained with PAR-CLIP experiments, the similarity with the clusters analyzed and described in the previous paragraphs is 0.78 (measured as the average Jaccard distance among corresponding clusters). After filtering for the CLIP, CLIP-seq, HITS-CLIP, and iCLIP (other-CLIP) interaction data, too few RBPs (12) were left in the dataset to extract a reasonable number of clusters, so that no comparative analysis with the clusters obtained considering the whole set of interactions could be performed. Finally, the clusters extracted from the third dataset (RIP) had, on average, very few associated genes (83). We expect that this lack of large scale interaction data will be overcome once more datasets are made available.

Overall, these results suggests that, to date, the main component driving the selection of clusters is the set of interactions measured by PAR-CLIP experiments.

### 2 - Filtering targets and regulators by expression level

So far, we have used PTR*comb*iner with a dataset that comprised all the binding evidence from multiple experiments in different biological systems that shared only the “human” source. The biological interpretation of results would greatly benefit from combining interaction data with other sources of information, such as expression data when available, in order to generate more specific hypotheses. To integrate PTR*comb*iner with expression data, we introduced the possibility to filter interactions according to: a) the expression levels of mRNA targets; and b) the expression levels of the regulators. In this way, the interactions can be filtered according to the specificity of the biological system being studied.

To show this functionality, we chose a well-studied cell line (HeLa) and filtered the AURA dataset by selecting: a) the transcripts known to be highly expressed in HeLa (based on RNA-seq data from [53]); and b) RNA binding proteins and miRNA known to be present in HeLa (based on SILAC data from [53] and small RNA-seq data from [54], respectively). The filtered dataset now comprises 53,560 UTRs (79% of the whole set of 67,962 UTRs), 81 RBPs (76% of the whole set of 106 RBPs), and 133 miRNAs (29% of the whole set of 463 miRNAs). We ran PTR*comb*iner on this filtered dataset, with the optimal *τ* parameter corresponding to 0.65. The resulting clusters are displayed in Additional file 9: Table S2. The majority of the clusters are composed of RBPs, with the exception of six singleton clusters and three mixed clusters of miRNAs and RBPs. To analyze the effect of the cell-specific filter on the resulting clusters, we compared these clusters (termed HeLa clusters) with those obtained in the first analysis that lacked any gene expression-abased filter (termed general clusters). The comparison is shown in Additional file 9: Table S3, where each HeLa-cluster is matched to the general-cluster with maximal Jaccard similarity. The average Jaccard similarity was 0.73 and ranged from nearly identical clusters (with a maximum similarity of 0.93) to clusters sharing half of their members (with a minimum similarity of 0.50). Interestingly, one-third of the general clusters (excluding the singletons R04, R05, R07, R09, R10, R17, and R24) could not be associated with any HeLa cluster. This is not surprising since these clusters are mainly composed of trans-factors or corresponding targets that are not expressed in HeLa cells. HeLa clusters therefore represent a subset of the general clusters.

### 3 - Balancing the trans-factor sample size

**Figure 7:**
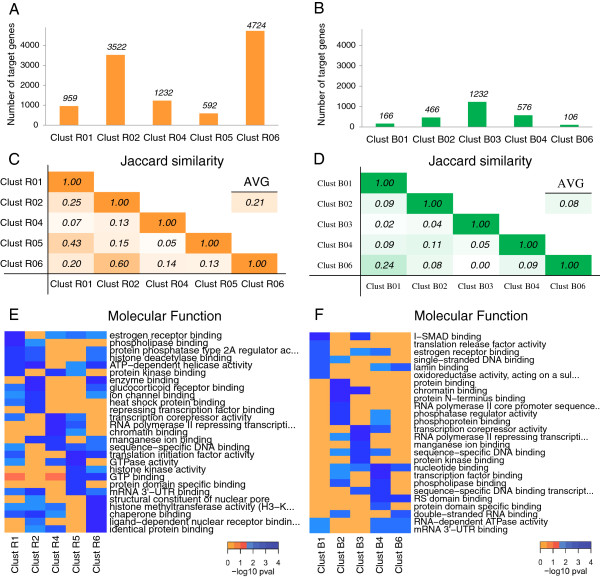
**Analyzing combinatorial features: comparison between unbalanced and balanced normalization.** The number of HGNC genes targeted by the top five ranking clusters obtained using unbalanced normalization **(A)** or balanced normalization **(B)** are displayed. Jaccard similarities among the target genes of the top five ranking clusters obtained with unbalanced normalization **(C)**, or those with balanced normalization **(D)** are shown. Heatmap showing the top enriched Molecular Function GO terms associated to the lists of genes targeted by the top five ranking clusters obtained with unbalanced normalization **(E)** or balanced normalization **(F)** are given. Singletons were excluded from the analyses.

An additional alternative workflow supported by PTR*comb*iner regards the mining step of the algorithm and arises from the previously discussed bias of the algorithm towards “widely interacting” trans-factors (see Mining combinatorial features). Introducing a balanced trans-factor association score is a possible way to reduce this bias. The difference between the standard version of PTR*comb*iner (called “unbalanced PTR*comb*iner”) and the proposed alternative workflow (called “balanced PTR*comb*iner”) is the way in which the pool of possible clusters is selected. The standard version of the algorithm incrementally extracts clusters of trans-factors by taking each candidate trans-factor as a “seed” and computing its association score with other trans-factors. The association score is the number of shared targets between the two factors, normalized by the number of targets of the seed (see Methods for details). This implies that the seed is required to have a significant fraction of targets in common with another trans-factor for that trans-factor to be included in the seed’s cluster. By construction, this score is asymmetric and tends to associate trans-factors with many interactions (e.g., AGO1) together with those with fewer interactions (which act as seeds) that share a significant fraction of targets with them. Here, we used a novel, alternative version of the association score normalized by the square root of the product of the target number for each trans-factor (cosine normalization, see Methods), thereby rebalancing the interaction data to cope with factors having a widely different number of targets. Note that the optimal threshold *τ* in this case is different from the unbalanced one (i.e. 0.25 vs 0.6), as the alternative association score strongly affects clusters sizes. Clusters obtained with the balanced score covered a larger number of trans-factors (88, comprising 39 RBPs and 49 miRNAs) than did the standard (unbalanced) version of the algorithm (with 56 trans-factors, comprising 32 RBPs and 24 miRNAs) (Table 1 and Additional file 9: Table S4, respectively). Although the average length of the clusters was the same, the balanced PTR*comb*iner produced more singleton clusters (namely, 12 out of 25, as compared to 5 out of 25 for the unbalanced) and a few very large clusters. Intuitively, the larger the cluster, the fewer its associated genes (as they need to be targeted by all trans-factors in the cluster). For this reason, the number of genes associated to the non-singleton clusters in the balanced case is lower than that in the unbalanced case (Figure 7A,B). Also, the Jaccard similarity between clusters, calculated over their trans-factors, is lower in the balanced case (Figure 7C,D). Clusters in the balanced case are thus more specific. Indeed, heat maps of the enriched GO terms have less overlap with respect to the unbalanced case (Figure 7E,F). A possible drawback of the balanced workflow is a tendency to create clusters that pick elements with a similar number of interactions. This excludes for instance clusters with miRNAs and RBPs that emerge from the standard workflow even after removing recurrent trans-factors (e.g., PUM2 plus miRNAs). The two procedures thus have different characteristics, allowing users to discover different types of interesting combinatorial associations and to alternatively analyze the data according to their specific needs.

## Comparison to related work

PTR*comb*iner is a novel approach mining combinatorial post-transcriptional regulation patterns from interaction data at the genome-wide level. As mentioned in the Background, previous attempts have been made in recent years to develop automated approaches to identify combinatorial aspects of post-transcriptional regulation. Several computational tools have been developed for miRNA target prediction using a number of different approaches, such as TargetScan [55], miRanda [56], TargetBoost [57], PITA [58], MAGIA2 [29], miRTar Hunter [59]. Target site predictions for different miRNAs have been combined in the tools PicTar [30] and ComiR [31] to identify potential groups of interacting miRNAs. By using simple model systems such as *S. cerevisiae*, some published studies have attempted to develop computational and experimental methods that identify functional modules based on combinatorial (or concurrent) RNA-protein interactions. For example, Joshi and coworkers [33] developed a probabilistic method for inferring regulatory module networks from expression profiles. In the following, we provide a more in-depth comparison with these approaches, highlighting the differences with respect to our proposed method and reporting comparative quantitative analyses on benchmarks for which these alternative approaches can be run.

### 1 - Comparison to PicTar

PicTar is a probabilistic predictive method that computes the probability of multiple miRNAs co-binding to the same target mRNA by combining the binding scores for each candidate miRNA. Albeit focusing on combinatorial interactions, the algorithm differs from PTR*comb*iner in many aspects. First, it explores the combinatorial interaction of miRNAs only. Second, it relies on predictive methods for detecting potential binding sites rather than using experimental data. The predictive approach can be reasonably accurate for miRNAs but are still far from satisfactory for RBPs. The third and main caveat of the method is that it lacks a mining procedure that allows to efficiently search the combinatorial space of possible clusters. Rather, it either requires the user to specify a set of miRNA to be jointly evaluated, or it needs to try all possible combinations in order to identify the high scoring ones. PTR*comb*iner takes a different perspective, implementing a mining approach to efficiently explore the combinatorial space of candidate clusters of miRNA and/or RBP, guided by their coverage of observed interactions with the target mRNAs. As discussed in the Conclusions, we plan to adapt the method to deal with weighted interactions, e.g., the score of a predicted binding or the confidence of a certain experimental technique.

In order to quantitatively compare the two approaches, we analyzed clusters of miRNA found by PTR*comb*iner using PicTar. We focused on the clusters S09 and S14 (Table 2) because they are composed of only four miRNAs, and evaluating larger clusters with PicTar is too computationally expensive. For each cluster, we took the set of its target genes, i.e. the genes which interact with all miRNAs in the cluster, and computed the PicTar interaction score of each of them with the cluster (see Additional file 10). The score is computed as the maximum value of the product of the binding scores of the miRNAs in the cluster, with the constraint that binding sites of different miRNAs should not overlap. If this constraint cannot be satisfied, or one or more miRNAs do not have predicted binding sites on the target, the score was set to zero. Binding scores for miRNAs were downloaded from the Dorina database [60]. We then compared these cluster-target scores with those obtained by running the same procedure on the entire set of genes (12,713 genes found in the Dorina dataset) using the Welch’s two samples t-test. The results of the statistical tests are presented in Additional file 10. For both clusters, the difference between cluster-target scores and general scores was statistically significant, with a confidence of approximately 99%. The fact that the average on the former set is significantly higher than the average on the latter set is not a trivial information. In fact, it indicates that also PicTar estimated the clusters of trans-factors to be relevant exactly to the specific set of genes used to cluster them together, confirming the relevance of the clusters mined by PTR*comb*iner.

### 2 - Comparison to ComiR

ComiR [31] is a web tool for combinatorial miRNA target prediction. It uses miRNA expression levels in combination with thermodynamic modeling and machine learning techniques to make combinatorial predictions. ComiR sums up the weighted (according to expression levels) scores of the single miRNAs, computed according to the four different scoring schemes of miRanda [56], PITA [58], TargetScan [55], and mirSVR [61]. These scores are combined through a support vector machine (SVM), which outputs the likelihood that the set of miRNAs targets a specific gene. As for PicTar, the main difference with PTR*comb*iner is the lack of a mining procedure that would identify the clusters of miRNA to be evaluated.

Using ComiR, we analyzed all clusters composed only of miRNAs extracted by PTR*comb*iner, namely, S09, S14, S16, and S22 (Table 2). In computing scores, we gave no expression level to ComiR, resulting in uniform level for all miRNAs. As for the PicTar case, we compared cluster-target scores with scores for the entire set of genes, i.e. all genes in ComiR output (Additional file 10). Welch’s two sample tests confirmed the statistical significance of the difference between cluster-target and general scores for all clusters, with a confidence of approximately 100% (Additional file 10). Similarly to the comparison to PicTar, this result confirms the relevance of the clusters mined by PTR*comb*iner.

### 3 - Comparison to LeMoNe

LeMoNe [62,63] is a probabilistic method for inferring regulatory module networks from expression profiles. The module network model was introduced in the work by Segal et al. [32] as a probabilistic bi-clustering approach, extracting co-clusters of genes and conditions from a matrix of gene expression levels under different experimental conditions. A co-cluster contains a subset of genes and conditions, such that the genes have similar expression levels under the selected conditions. The subset of genes represents a regulatory module, while each subset of conditions for the same module is an expression state for the module. LeMoNe introduces an ensemble averaging strategy to generate more coherent modules from multiple runs of the module network inference process. The approach was later adapted to infer transcriptional and post-transcriptional modules from both transcriptome and translatome expression profiles in *S. cerevisiae*[33]. As already discussed, this approach detects putative regulatory modules that characterize specific biological conditions (i.e. stress conditions), while PTR*comb*iner targets more general purpose, genome-wide combinatorial interactions. Nonetheless, it is interesting to study the relationship between clusters detected by the two methods, when both are applicable (i.e. if the translatome expression profiles are available). To compare this method with PTR*comb*iner, we ran our computational tool on the yeast dataset employed in [33].

**Figure 8:**
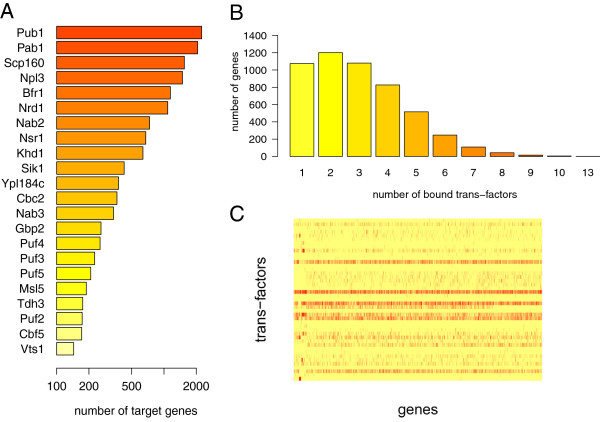
**Interaction maps annotated for*S. cerevisiae*.(A)** Yeast trans-factors (41 RBPs) were ordered according to the number of their annotated target genes. Trans-factors with less than 100 distinct targets are not shown. **(B)** Distribution of the number of distinct trans-factors bound to the same gene. **(C)** Graphical representation of the Boolean interaction matrix, derived from the input pairwise interactions. Each row corresponds to a trans-factor, each column to a gene. Positive interactions are displayed in red.

The interaction dataset [64] contains RIP-chip experiments involving 43 RBPs and 5,118 genes. We obtained a list of interacting RBPs and their relative number of target genes (Figure 8A). The distribution of the number of distinct trans-factors bound to the same gene are shown in Figure 8B. In total, the dataset contains 15,391 annotated interactions, with the interaction matrix sparsity value of 0.07 (Figure 8C). From the interaction matrix, PTR*comb*iner (with optimal *τ* set to 0.4) extracted the clusters composed exclusively of RBPs (Additional file 9: Table S5). These clusters were then compared with the sets of RBPs whose targets were enriched in post-transcriptional modules generated by LeMone. The list of RBP sets was extracted from the supplementary materials of [33] (Additional file 9: Table S6).

PTR*comb*iner clusters were then compared with LeMoNe clusters, according to the Jaccard similarity maximized by matching each PTR*comb*iner cluster with a LeMoNe cluster (Additional file 9: Table S7). Reassuringly, the agreement between the two methods is high, with half of the top ten PTR*comb*iner clusters identical to LeMone clusters.

## Conclusions

In this paper we present a computational tool for the combinatorial analysis of post-transcriptional regulation patterns involving multiple trans-factors. This tool, called PTR*comb*iner, was tested on two sets of experimental interactions between post-transcriptional trans-factors and target mRNAs, in human and yeast. PTR*comb*iner enables the user to: a) detect groups of regulators that share a conspicuous amount of mRNA targets; b) characterize the clusters biologically; and c) identify concurrent binding sites of trans-factors belonging to the same cluster. Further, the underlying Boolean matrix factorization approach allows the user to identify multiple overlapping clusters of trans-factors that jointly account for as many interactions as possible, casting the problem into an approximate interaction coverage task. This method naturally addresses the limitations of most clustering and motif mining approaches, which typically return non-overlapping clusters (of trans-factors), clusters covering non-overlapping sets (of mRNAs), or long lists of highly redundant clusters. Our tool is an original and comprehensive attempt to provide a computational pipeline for elucidating complex post-transcriptional combinatorial rules on a genome-wide level. By integrating expression profiles of both trans-factors and target mRNAs, the tool can also be used to mine combinatorial patterns in specific experimental conditions. We plan to extend the method to deal with uncertainty in the interaction information, such as putative interactions from predictive algorithms, and to incorporate the binding strength by properly weighting the contribution of each interaction.

Importantly, PTR*comb*iner is a versatile tool that is not limited to post-transcriptional regulation analysis. In fact, it can be easily adapted to mine transcriptional and combined transcriptional/post-transcriptional regulation patterns. An inherent limitation in the analyses that can be conducted with the tool is given by the paucity of interaction data available to date. However, given the fast rate at which high-throughput interaction detection experiments are conducted, this tool may have the potential to unveil the complex mosaic of interactions underlying post-transcriptional regulation in the near future.

## Methods

### Data extraction

Interaction data used to build the human interaction matrix and to classify RBP binding sites were extracted from the AURA 2 database (/http://aura.science.unitn.it/download/). AURA 2 collects experimental post-transcriptional interactions from different techniques as annotated in published literature, storing also positional binding information if available, without introducing additional data manipulation steps (apart from mapping coordinates to the hg19 genome assembly).

Interaction data used to build the yeast interaction matrix were extracted from [64].

### Boolean matrix factorization

The Mining module factorizes a *n*×*m* Boolean matrix *C* representing the interaction maps in the available dataset. Formally:

$$C_{\text{ij}}=\left\{\begin{array}{cc} 1 & \;\;\text{if trans-factor}\;j\;\text{interacts with target}\;i\\ 0 & \;\;\text{otherwise} \end{array}\right)$$

For example, in the case of the interaction maps taken from AURA 2 [35], the interaction matrix *C* represents trans-factor—UTR interactions (where trans-factors can be either RBPs or miRNAs), while in the case of interaction maps on yeast [64], it represents RBP-gene interactions. Even so, the mining module can analyze any dataset containing interaction maps.

The mining module uses the Boolean matrix factorization algorithm developed by Miettinen et al. [36] to identify clusters of trans-factors which bind the same set of targets. Let *m* be the number of different trans-factors, and *n* the number of targets. Let *C* be a *n*×*m* Boolean matrix which represents trans-factor—target interactions. The rows of the matrix (observations) represent the targets, and the columns (attributes) represent the trans-factors. A *basis vector* represents a set of correlated attributes, i.e. a cluster of trans-factors. Boolean matrix factorization aims to discover the clusters of trans-factors that are present in the dataset and how the interactions of each target can be expressed by a combination of these clusters.

Let *S* and *B* be binary matrices of dimensions *n*×*k* and *k*×*m*, respectively. The *n*×*m* matrix *S*∘*B* represents the Boolean product of *S* and *B*, with the addition defined as 1+1=1. In a more intuitive way, *B* is the *basis vector matrix* that contains the information about which trans-factors appear in each cluster, and *S* is the *usage matrix* that contains the information about which clusters of trans-factors appear in each target.

Given the binary *n*×*m* interaction matrix *C* and a positive integer *k*≤ min{*n*,*m*}, the *Boolean matrix factorization* procedure finds an *n*×*k* matrix *S* and a *k*×*m* binary matrix *B* that minimize

$$\left|C-S\circ B|=\overset{n}{\underset{i=1}{\sum }}\overset{m}{\underset{j=1}{\sum }}|C_{\text{ij}}-{\left(S\circ B\right)}_{\text{ij}}\right|$$

Since the exact factorization of the matrix *C* is an NP-hard problem, the algorithm greedily builds an approximate solution to the factorization problem. It constructs the basis matrix *B* (and accordingly, the usage matrix *S*) to try to cover the ones in the interaction matrix *C* in a greedy manner, giving the priority to the denser rows of the matrix (with a high proportion of ones). The basic idea behind the greedy algorithm is to exploit the correlation between the columns (the trans-factors). First, the associations between pairs of trans-factors are computed and used as candidate basis vectors. Second, *k* of these basis vectors are selected in a greedy fashion. Let *A* be an *m*×*m* Boolean matrix that contains *m* candidate basis vectors. *A*_*ij*_=1 if the correlation between trans-factor *i* and trans-factor *j* is *τ*-strong, which means that it is no less than a certain threshold value *τ*≤1, and 0 otherwise.

In this work, two different approaches to estimate the association score between trans-factors are presented. The standard version of the algorithm [36] uses an *unbalanced* score, i.e. the association of the *i*-th trans-factor with the *j*-th one is defined as *c*(*i*⇒*j*)=〈**c**_.*i*_,**c**_.*j*_〉/〈**c**_.*i*_,**c**_.*i*_〉, where 〈·,·〉 is the inner product between vectors, and in general *c*(*i*⇒*j*)≠*c*(*j*⇒*i*). The resulting association matrix is an *m*×*m* asymmetric Boolean matrix. The *i*-th row of *A*, which corresponds to the *i*-th candidate basis vector (cluster) is determined using the *i*-th trans-factor as seed. This means that *A*_*ij*_=1 if the number of common targets between the *i*-th and the *j*-th trans-factor is at least a fraction *τ* of the number of targets of the *i*-th trans-factor, and 0 otherwise. The score is thus normalized only according to the number of targets of the seed trans-factor. As a consequence, trans-factors with many targets tend to have a high association score with many trans-factor seeds, when these have few interactions, and thus to appear in multiple clusters. This allows to identify combinatorial interactions between trans-factors with different degree of specificity (e.g. RBPs and miRNAs). On the other hand, clusters of purely specific trans-factors tend to be discarded by the selection procedure, which aims at maximizing the interaction coverage.

To address this bias, we implemented an alternative *balanced* association score given by the vector cosine similarity, i.e. $c(i\Leftrightarrow j)=\langle c_{\mathrm{.i}},c_{\mathrm{.j}}\rangle /\sqrt{\langle c_{\mathrm{.i}},c_{\mathrm{.i}}\rangle \cdot \langle c_{\mathrm{.j}},c_{\mathrm{.j}}\rangle }$. The resulting association matrix is symmetric and produces more homogeneous clusters in terms of the number of targets of their trans-factors.

The two association scores have different characteristics, allowing the discovery of different types of interesting combinatorial associations.

### Biological characterization

#### Jaccard similarity

The Jaccard similarity between two lists is defined as the ratio between the size of the intersection and the size of the union of the two lists. This measure ranges from 0 (i.e. the two lists do not have any common element) to 1 (i.e. the two lists are identical).

#### Gene ontology enrichment analysis

Gene Ontology enrichment analysis was performed with the bioconductor package topGO (http://www.bioconductor.org/packages/2.13/bioc/html/topGO.html), using the Fisher’s exact test statistics and the “elim” method for dealing with the GO graph structure. The significance of over-representation is determined at a 0.05 P value threshold. Enrichment analysis was performed on the list of HGNC genes regulated by each cluster of trans-factors. Inside each cluster, enrichment analysis was performed also on the list of HGNC genes interacting with each trans-factor, in order to compare enrichments associated with targets of single trans-factors with enrichments associated to targets of clusters.

#### Semantic similarity

Semantic similarity between two lists of enriched GO terms was calculated with the bioconductor package GOsemsim [65] (http://bioconductor.org/packages/2.12/bioc/html/GOSemSim.html), using Wang’s method to calculate pairwise semantic similarities between GO terms and the BMA (best-match average) method to combine semantic similarity scores of multiple GO terms.

### RBP-binding site classifier

Often, RBP-binding sites are modeled taking into account only sequential information. In contrast, we acknowledge here that the distribution of regions available for interaction with RBPs is significantly influenced by the presence of self-interacting base pairs on the surrounding mRNA region. Moreover, RBPs are known that exhibit a specific binding preference for double-stranded RNA. Our key idea was to model interaction sites as RNA sequences that are free to self-interact and fold into stable structures. We therefore needed to: 1) reliably compute the folded structure of a mRNA sequence; and 2) develop predictive models that can accept such complex structures as input. For these reasons, our RBP Site Pairwise Classification module is implemented as a kernel machine binary classifier [50] using a graph kernel on predicted RNA secondary structures [34].

Kernelized learning algorithm is a popular machine learning approach in which the development of a suitable similarity function, called the *kernel*, allows computations to be performed over arbitrary data structures. Specifically, graph kernels allow predictive algorithms, like Support Vector Machines (SVM), to operate over graph instances. Using a machine learning method that is designed to work on data structures that are as flexible as graphs allows us to model the structure of RNA in a natural way, with vertices representing nucleotides and edges representing the different types of bonds between nucleotides, i.e. backbone phosphate bonds and base-pairing bonds. Moreover, recent advances in graph kernels [34] coupled with fast stochastic algorithms [66] allow the learning problem to be scaled to datasets comprising hundreds of thousands graphs, paving the way to *-omics* applications.

The core idea for the graph kernel that we use (called Neighborhood Subgraph Pairwise Distance Kernel or NSPDK in short) [34] is to generalize *(gapped) k-mers* string kernels to graphs. Instead of determining the similarity between two strings measuring the fraction of common k-mers (i.e. small contiguous subsequences), we determined here the similarity between two graphs by counting the shared fraction of a special type of compact subgraphs, called neighborhood subgraphs. A neighborhood subgraph is induced by all vertices which are at a distance not greater than a specified radius from a given root vertex (where the distance between two vertices is taken as the length of the shortest path between the vertices). Since checking whether two graphs are identical is more difficult than checking whether two strings are identical, an efficient approximation was proposed in [34], based on hashing a quasi-canonical graph representation.

In [67], the authors have shown how to apply NSPDK to a graph representation of RNA folding structures. The key idea is to not rely only on a single structure (e.g., the minimum free energy configuration), which is known to be error prone, but rather use efficient dynamic programming algorithms [68] to sample the set of all possible structures for the given sequence, taking a small number of representatives that are both structurally diverse and energetically stable. Finally, all of the structures relative to a single RNA sequence are considered simultaneously in a comprehensive disconnected graph.

The RBP Site Pairwise Classification module uses all these ideas in a unified framework: given a region of the mRNA, 1) a sample of highly stable but diverse folding structures is computed and encoded in a graph [67]; this graph is then 2) processed by the NSPDK kernel [34] and a corresponding feature representation is extracted; and 3) finally, binding sites of different RBPs are discriminated via a SVM which takes in input the mRNA regions encoded in the aforementioned feature representation. The SVM model is efficiently trained using the stochastic gradient descent technique of [66].

### Performance measures

The performance of classification tasks can be evaluated through a variety of values. Each example in the dataset has an observed label (positive or negative in the case of binary classification) that represents its actual class, and a predicted label (again, either positive or negative) that is predicted by the classifier. Comparing these two labels makes it possible to define the *True Positives* (TP) as the positive examples that were predicted as positive, the *True Negatives* (TN) as the negative examples that were predicted as negative, the *False Positives* (FP) as the negative examples that were predicted as positive, and the *False Negatives* (FN) as the positive examples that were predicted as negative. The *Precision* value is the True Positive rate, which is the fraction of True Positives with respect to the total amount of examples predicted as positive, while the *Sensitivity* is the fraction of TP with respect to the total amount of positive examples. The two measures are complementary, so that an increase in one typically results in a decrease in the other. The *F1-score* is defined as the harmonic mean between precision and sensitivity, trading off the two. This measure requires the classifier to output a hard decision for each example, i.e. either a positive or a negative prediction. Many classifiers provide a confidence for their predictions, so that a user can choose a threshold over which a prediction is considered as positive. By varying the threshold one can obtain a spectrum of predictions, from very conservative (only the most confident predictions are positive) to very tolerant. The *AUROCC* (Area Under the Receiver Operative Characteristic Curve) is an aggregate measure evaluating the performance of a classifier over the whole spectrum of possible thresholds. It is obtained by plotting the TP rate vs the FP rate (i.e. the fraction of negative examples predicted as positive) when varying the threshold value, and computing the area under the resulting curve. An AUROCC value of 0.5 indicates that the classifier is completely unable to discriminate between the two classes, performing as a random predictor, while an AUROCC value of 1 indicates perfect discrimination.

## Availability of supporting data

The data sets supporting the results of this article are included within the article (and its Additional files 1 and 8).

## Abbreviations

PTR: Post-transcriptional regulation; UTR: Untranslated region of mRNA; RBP: RNA-binding protein; ncRNA: Non-coding RNA; CLIP: Cross-linking and immunoprecipitation; AURA: Atlas of UTR Regulatory Activity; BMF: Boolean matrix factorization; NSPDK: Neighborhood subgraph pairwise distance kernel; AUROCC: Area under the receiver operating characteristic curve.

## Competing interests

The authors declare that they have no competing interests.

## Authors’ contributions

GC implemented the tool; TT implemented the tool and prepared the figures; GB implemented the first prototype of the mining module; FC implemented the RBP-site classifier; AQ conceived the work; GV conceived and supervised the work, produced Figure 1; AP conceived and supervised the work and contributed to the final implementation of the tool. GC, TT, FC, AQ, GV and AP wrote the paper. All authors read and approved the final manuscript.

## Supplementary Material

Additional file 1 **A zip archive containing the interaction map sets used to discuss the performance of PTR** ***comb*** **iner in this paper.** Click here for file

Additional file 2 **A zip archive containing the outputs of the mining module of PTR** ***comb*** **iner at different combinations of** ***k* and** ***τ*, including recurrent trans-factors.** Click here for file

Additional file 3 **A zip archive containing the outputs of the mining module of PTR** ***comb*** **iner at different combinations of** ***k* and** ***τ*, considering the sporadic trans-factors.** Click here for file

Additional file 4 A table of two columns containing the associations between HGNC genes and clusters of trans-factors obtained including recurrent trans-factors. Click here for file

Additional file 5 A table of two columns containing the associations between HGNC genes and clusters of sporadic trans-factors. Click here for file

Additional file 6 **A zip archive containing the ontological enrichment results generated by PTR** ***comb*** **iner analyzer module step 1 for the top five ranking clusters obtained including recurrent trans-factors.** Click here for file

Additional file 7 **A zip archive containing the ontological enrichment results generated by PTR** ***comb*** **iner analyzer module step 1 for the top five ranking clusters of sporadic trans-factors.** Click here for file

Additional file 8 A zip archive containing the binding site coordinates for all the RNA binding proteins annotated in AURA 2. Click here for file

Additional file 9 A zip file containing supplementary Figure S1 and supplementary Tables S1–S7. Click here for file

Additional file 10 A zip archive containing the PicTar and ComiR scores used for confrontation with our method. Click here for file
