# Supplementary material for: PTRcombiner: mining combinatorial regulation of gene expression from post-transcriptional interaction maps
Source: BMC Genomics. 2014 Apr 23;15:304. doi: 10.1186/1471-2164-15-304 (PMC4234518; doi:10.1186/1471-2164-15-304)
Supplement: Additional file 10 — A zip archive containing the PicTar and ComiR scores used for confrontation with our method. [file 1471-2164-15-304-S10.zip › Supplementary Figure S1.pdf]

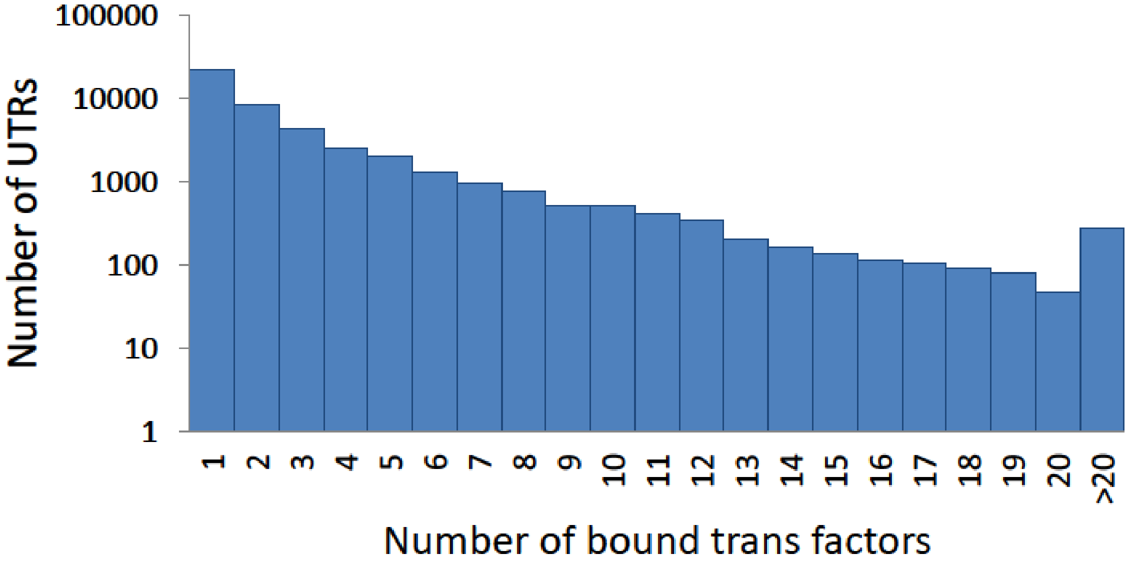

Figure S1: Distribution of the number of distinct trans-factors bound to the same UTR when removing recurrent trans-acting factors from the analysis. The distribution ranges from 1 to 53, with mean 3.
